# Child maltreatment in Germany: prevalence rates in the general population

**DOI:** 10.1186/s13034-017-0185-0

**Published:** 2017-09-29

**Authors:** Andreas Witt, Rebecca C. Brown, Paul L. Plener, Elmar Brähler, Jörg M. Fegert

**Affiliations:** 1 Department of Child and Adolescent Psychiatry/Psychotherapy, University of Ulm, Steinhövelstr. 5, 89073 Ulm, Germany; 2 Department of Psychosomatic Medicine and Psychotherapy, University Medical Center of Johannes Gutenberg University Mainz, Mainz, Germany; 3 Department of Medical Psychology and Medical Sociology, University of Leipzig, Leipzig, Germany

**Keywords:** Prevalence, Child maltreatment, Child abuse and neglect, Representative study

## Abstract

**Background:**

Child maltreatment and its consequences are considered a major public health problem. So far, there is only one study from Germany reporting prevalence rates on different types of maltreatment.

**Methods:**

A representative sample of the German general population was examined for experiences of child maltreatment using the Childhood Trauma Questionnaire (CTQ) between September and November 2016. A total of 2510 (53.3% female) participants between 14 and 94 years (M = 48.8 years) were enrolled. Besides the CTQ, a range of sociodemographic information was collected. The interrelatedness of different types of maltreatment was examined using configuration analysis and predictors for maltreatment were identified by performing binary logistic regression analyses.

**Results:**

Overall, 2.6% (f: 3.9%, m: 1.2%) of all participants reported severe emotional abuse, 3.3% (f: 3.4%, m: 3.3%) severe physical abuse, 2.3% (f: 3.7%, m: 0.7%) severe sexual abuse, 7.1% (f: 8.1%, m: 5.9%) severe emotional neglect and 9% (f: 9.2%, m: 8.9%) severe physical neglect. Women were more likely to report at least moderate sexual and emotional abuse than men. The largest difference between age groups was reported for physical neglect, with participants aged over 70 years reporting the highest rates. Participants who reported childhood maltreatment were more likely to be unemployed or have lower educational outcomes. The most common combination of maltreatment types were physical and emotional neglect, all five types of maltreatment combined and physical and emotional neglect and physical abuse combined.

**Conclusions:**

Child maltreatment, especially physical neglect is common in the German population. Women seem to be at greater risk for sexual and emotional abuse than men. Knowledge about different types of maltreatment based on the Childhood Trauma Questionnaire (CTQ) can help to put findings of future studies into an epidemiological and societal context.

## Background

Child maltreatment is considered a major public health problem [1–3]. The consequences of maltreatment are diverse and may affect victims throughout their whole lifespan via psychological and behavioral problems, as well as somatic disorders [1, 4–11]. As a consequence, in addition to these individual consequences, maltreatment causes high financial burden for society. Previous studies estimated annual expenses caused by maltreatment between 11 to 30 billion Euros for Germany [7] and up to 124 billion US Dollars per year in the US [8].

Results from international studies show that child maltreatment is highly prevalent. This is also true in high income countries where prevalence rates are comparable to those of widespread diseases [9, 10]. Meta-analyses on the prevalence of different types of maltreatment exist, and especially child sexual abuse having been reviewed repeatedly [10–13]. One meta-analyses showed varying prevalence rates mostly due to varying definitions, but also due to methodological factors, like small sample sizes, geographical regions or non-random designs [11, 13]. One review by Stoltenborgh and colleagues [11] focused on the assessment of child sexual abuse in adult populations and included 331 independent samples with a total of almost 10 million participants. The overall prevalence for self-reported child sexual abuse was reported at around 12.7% (95% confidence interval (CI) 10.7–15.0%), 18% for women and 7.6% for men. These rates are comparable to those found in other meta-analyses [10, 12] and also in a meta-analysis focusing on prevalence rates of sexual abuse in adolescent populations [13]. Overall, females seem to be more often affected by sexual abuse than men.

For other types of maltreatment like neglect, the data base is less comprehensive. The so-called “neglect of neglect” is still evident in research [3, 14, 15]. Meta-analyses on physical and emotional abuse, as well as neglect show a high variation in prevalence rates [15–17]. Regarding physical abuse, prevalence rates of 22.6% (95% CI 19.6–26.1%) were reported by Stoltenborgh and colleagues [16], similar to 22.9% by Sethi and colleagues for the European Region [10]. Larger differences were reported for emotional abuse with a rate of 36.3% worldwide [17] and 29.1% reported from the European Union [10]. With regard to child neglect, Stoltenborgh and colleagues identified 19 independent samples, underlining the need for further studies on neglect. Prevalence rates were reported at 16.3% (95% CI 12.1–21.5) for physical and 18.4% (95% CI 13.0–25.4) for emotional neglect. In contrast to findings on sexual abuse, there does not seem to be a gender preponderance for the other types of maltreatment [3, 15–17].

For Germany, data on the prevalence of child maltreatment in the general population is limited to three data sets: two studies, which were conducted almost 20 years apart from each other, focused on the assessment of child sexual abuse [18–20]. They reported a marked decline of sexual abuse over a period of almost 20 years. Only one study reported on the prevalence of different types of maltreatment in the general population. The study was conducted in 2010 using the Childhood Trauma Questionnaire (CTQ) [21, 22] and reported a prevalence of 1.6% for severe emotional, 2.8% for severe physical, 1.9% for severe sexual abuse, and 6.6% for severe emotional and 10.8% for severe physical neglect [22].

In summary, data on the prevalence of different types of child maltreatment exist, however usually only general prevalence rates for different types of maltreatment and males and females are reported. On closer examination, prevalence rates vary considerably across different subgroups (e.g. age cohorts or gender) [18, 22]. As the CTQ has been used in a range of brain imaging studies as a covariate [23, 24] and it is a widely used screening instrument for the assessment of child maltreatment [22, 25, 26] recent data need to be made available to set new scientific findings into context and inform the debate of societal burden by childhood maltreatment.

The aim of the present study is to provide recent and detailed prevalence rates for all types of maltreatment as assessed by the CTQ (emotional, physical, and sexual abuse, as well as physical and emotional neglect) in a representative sample of the general population in Germany.

## Methods

### Procedure

Data collection took place between September and November 2016. Using a random route procedure, a representative sample of the German population was obtained by a demographic consulting company (USUMA, Berlin, Germany). The sample was representative in regard to age, gender, and geographic region. Households of every third residence in a randomly chosen street were invited to participate in the study. In multi-person households, participants were randomly selected using a Kish-Selection-Grid. For inclusion, participants had to be at least 14 years of age and have sufficient German language skills. Of 4902 designated addresses, 2510 households participated in the study. The main reason for non-participation was failure to contact anyone in the residence after four attempts (14.9%), refusal by the individual who answered the door to have anyone in the household participate in the study (15.3%), failure to contact the randomly selected household member after four attempts (2.3%) and refusal by the selected member to participate (14.7%).

Individuals who agreed to participate were given information about the study and provided informed consent. Participants were told that the study was about psychological health and well-being. Responses were anonymous. In a first step, socio-demographic information was obtained in an interview-format by the research staff. Then, the researcher handed out a copy of the questionnaire and a sealable envelope. The researcher remained nearby in case the participants needed further information. The completed questionnaires were linked to the respondent’s demographic data, but did not contain name, address, or any other identifying information.

The study was conducted in accordance with the Declaration of Helsinki, and fulfilled the ethical guidelines of the International Code of Marketing and Social Research Practice of the International Chamber of Commerce and of the European Society of Opinion and Marketing Research. The study was approved by the Ethics Committee of the Medical Department of the University of Leipzig.

### Measures

The sociodemographic section contained information on age, gender, citizenship, geographical area (East vs. West Germany, rural vs. urban area), educational and occupational status and partnership status. Additionally, an estimation of the equivalence income (household income divided by the square root of household size), according to OECD [27] was calculated.

The prevalence of five types of child maltreatment was assessed using the 28 item brief version of the Childhood Trauma Questionnaire (CTQ) [21, 28, 29]. The CTQ is a screening measure for the assessment of child maltreatment. The CTQ contains five subscales each assessed by 5 items, including sexual, emotional and physical abuse as well as emotional and physical neglect. Additionally, three items assess whether participants tend to minimize problematic experiences within their family. The psychometric properties of the German version of the CTQ have been demonstrated by Klinitzke and colleagues [21]. The internal consistency ranged between 0.62 and 0.96 for the subscales. The intra-class coefficient for an interval of 6 weeks was 0.77 for the overall scale and for subscales between 0.58 and 0.81. Based on norm data by Häuser and colleagues [22] severity scores for each subscale can be calculated, ranging from “none–minimal”, “minimal–moderate”, “moderate–severe”, to “severe–extreme”. For the prevalence analysis of the different types of maltreatment, a cut-off of at least “moderate–severe” was chosen.

### Participants

A total of 2510 participants were included in the sample. Participants were on average 48.4 years old (SD = 18.2) and 53.3% were female. 3.2% reported a place of birth outside Germany. The sample was representative for the German population in regard to age and gender. The sociodemographic characteristics are presented in Table 1.

**Table 1:** Demographic data

	Total (N = 2510)	Female (N = 1339)	Male (N = 1171)
Age			
Mean (standard deviation)	48.4 (18.2)	48.9 (18.1)	47.8 (18.4)
Range	14–94	14–94	14–93
Living with partner			
Yes	1370 (55%)	719 (54%)	651 (56.2%)
No	1119 (45%)	612 (46%)	507 (43.8%)
Citizenship			
German	2429 (96.8%)	1303 (97.3%)	1126 (96.2%)
Not German	81 (3.2%)	36 (2.7%)	45 (3.8%)
Geographical area			
Eastern Germany	505 (20.1%)	255 (19%)	921 (78.7%)
Western Germany	2005 (79.9%)	1084 (81%)	250 (21.3%)
Rural	1026 (40.9%)	548 (40.9)	478 (40.8%)
Urban	1484 (59.1%)	791 (59.1%)	693 (59.2%)
Occupational status			
Full-time	1074 (42.8%)	407 (30.4%)	667 (57%)
Part-time	281 (11.2%)	246 (18.4%)	35 (3%)
Hourly	60 (2.4%)	54 (4%)	6 (0.5%)
Federal volunteer service/parental leave	25 (1%)	22 (1.6%)	3 (0.3%)
Unemployed	131 (5.2%)	64 (4.8%)	67 (5.7%)
Retiree	638 (25.4%)	368 (27.5%)	270 (23.1%)
Homemaker	79 (3.1%)	77 (5.8%)	2 (0.2%)
In training	42 (1.7%)	21 (1.6%)	21 (1.8%)
Student	161 (6.4%)	70 (5.2%)	91 (7.8%)
Employment status			
Unemployed	131 (5.3%)	64 (4.8%)	67 (5.8%)
Employed	2360 (94.7%)	1265 (95.2%)	1095 (94.2%)

### Statistical analyses

All analyses were conducted using SPSS version 21. Descriptive analyses were conducted for prevalence rates. Comparisons were conducted using χ^2^ tests. To assess the co-occurrence of different types of child maltreatment a configuration analysis was conducted. Binary logistic regression analyses were conducted to identify predictors of childhood maltreatment. Age and gender were entered in the analyses as potential predictors.

## Results

Of the N = 2487 participants who completed the CTQ, 31.0% (n = 772) reported at least one type of child maltreatment. Of all participants, 6.5% reported at least moderate emotional abuse, 6.7% reported physical abuse, 7.6% sexual abuse, 13.3% emotional neglect, and 22.5% reported physical neglect (for details see Table 2).

**Table 2:** Prevalence of child maltreatment by severity

	N	None–minimal N (%)	Low–moderate N (%)	Moderate–severe N (%)	Severe–extreme N (%)
Emotional abuse					
Total	2492	2027 (80.8)	302 (12.0)	98 (3.9)	65 (2.6)
Female	1324	1053 (79.5)	156 (11.8)	64 (4.8)	51 (3.9)
Male	1168	974 (83.4)	146 (12.5)	34 (2.9)	14 (1.2)
Physical abuse					
Total	2497	2185 (87.1)	145 (5.8)	83 (3.3)	84 (3.3)
Female	1330	1165 (87.6)	79 (5.9)	41 (3.1)	45 (3.4)
Male	1167	1020 (87.4)	66 (5.7)	42 (3.6)	39 (3.3)
Sexual abuse					
Total	2496	2148 (85.6)	158 (6.3)	133 (5.3)	57 (2.3)
Female	1329	1090 (82.0)	89 (6.7)	101 (7.6)	49 (3.7)
Male	1167	1058 (90.7)	69 (5.9)	32 (2.7)	8 (0.7)
Emotional neglect					
Total	2496	1486 (59.2)	678 (27.0)	155 (6.2)	177 (7.1)
Female	1329	809 (60.9)	334 (25.1)	78 (5.9)	108 (8.1)
Male	1167	677 (58.0)	344 (29.5)	77 (6.6)	69 (5.9)
Physical neglect					
Total	2496	1452 (57.8)	482 (19.2)	336 (13.4)	226 (9.0)
Female	1329	786 (59.1)	251 (18.9)	170 (12.8)	122 (9.2)
Male	1167	666 (57.1)	231 (19.8)	166 (14.2)	104 (8.9)

### Co-morbidity of types of child maltreatment

Overall, 58.10% (N = 416) of those reporting any form of child maltreatment reported only one type of maltreatment. In detail, 47.15% (N = 265) of those reporting physical neglect (N = 562) 31.58% (N = 60 out of N = 190) of those reporting sexual abuse, 14.76% (N = 49 out of N = 332) of those reporting emotional neglect, 13.17% (N = 22 out of N = 167) of those reporting physical abuse, and 12.27% (N = 20 out of 163) of those reporting emotional abuse did not report another type of maltreatment. The most common combination of types of child maltreatment were physical and emotional neglect (13.99%), all five types of maltreatment combined (3.89%) and physical and emotional neglect and physical abuse combined (3.50%) (for details see Table 3).

**Table 3:** Prevalence of different types and combinations of maltreatment

Type/combination of maltreatment	N	Percent in relation to participants with at least one type of maltreatment (N = 772)
Emotional abuse (EA) only	20	2.59
Physical abuse (PA) only	22	2.85
Sexual abuse (SA) only	60	7.77
Emotional neglect (EN) only	49	6.35
Physical neglect (PN) only	265	34.33
EA + PA	4	0.52
EA + SA	12	1.55
EA + EN	13	1.68
EA + PN	5	0.65
PA + SA	1	0.13
PA + EN	3	0.39
PA + PN	11	1.42
SA + EN	3	0.39
SA + PN	13	1.68
EN + PN	108	13.99
EA + PA + SA	3	0.39
EA + PA + EN	18	2.33
EA + PA + PN	4	0.52
EA + SA + EN	4	0.52
EA + SA + PN	3	0.39
EA + EN + PN	7	0.91
PA + SA + EN	1	0.13
PA + SA + PN	4	0.52
PA + EN + PN	27	3.50
SA + EN + PN	20	2.59
EA + PA + SA + EN	2	0.26
EA + PA + SA + PN	10	1.30
EA + PA + EN + PN	21	2.72
EA + SA + EN + PN	6	0.78
PA + SA + EN + PN	14	1.81
EA + PA + SA + EN + PN	30	3.89

*EA* emotional abuse, *PA* physical abuse, *SA* sexual abuse, *EN* emotional neglect, *PN* physical neglect

### Predictors of moderate to severe types of maltreatment

**Fig. 1:**
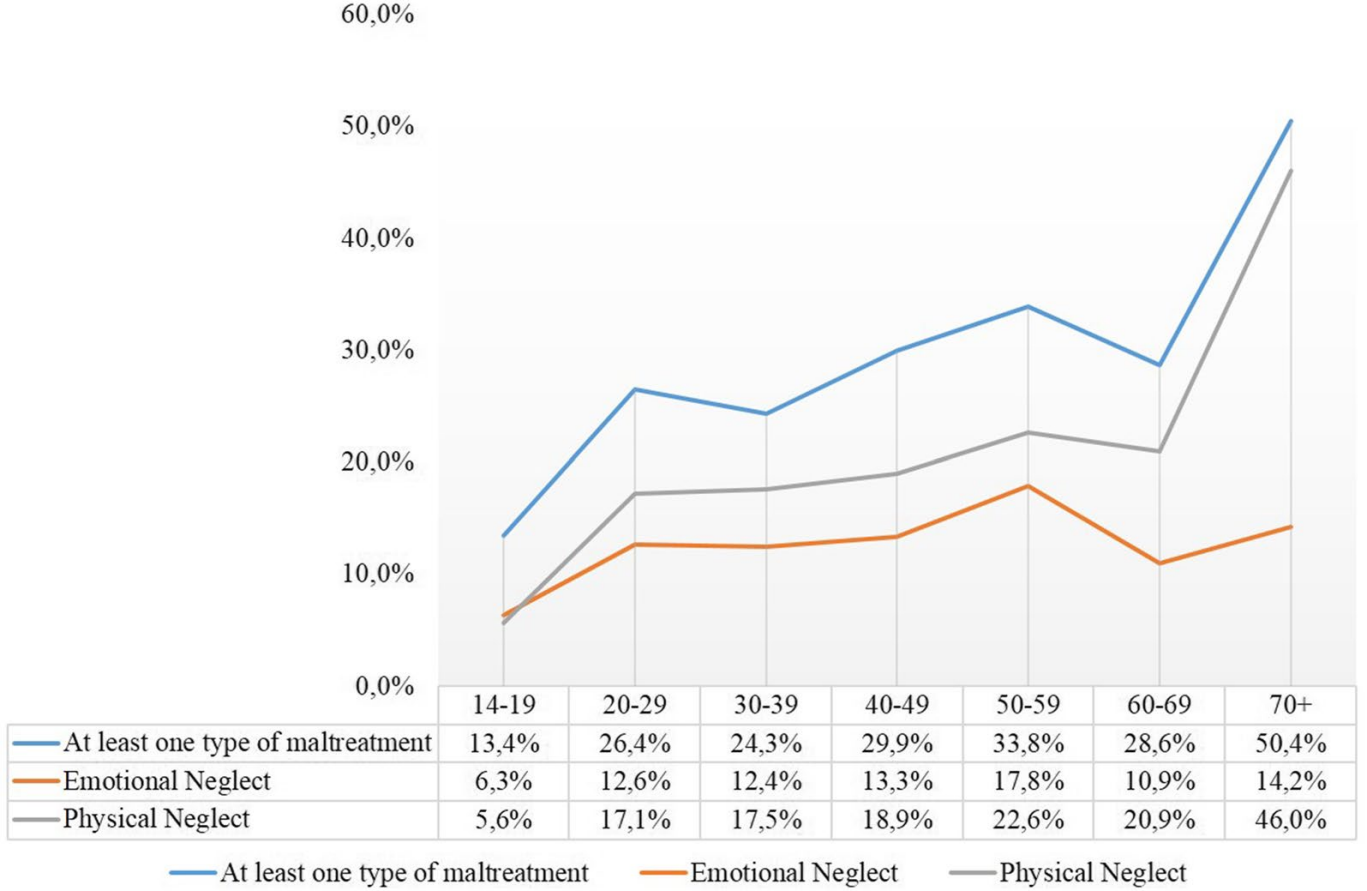
Prevalence of child neglect and having experienced at least type of maltreatment divided by age groups

Gender was shown to be a predictor for emotional and sexual abuse, with women reporting higher rates of both types of abuse (see Table 4; details on gender differences by severity of maltreatment are also presented in Table 2). Furthermore, age was identified as a predictor for physical neglect, with higher age being associated with higher prevalence rates (see Table 4; Fig. 1 for details).

**Table 4:** Binary logistic regressions for predictors of different types of maltreatment

Dependent variable	Independent variable	Odds ratio (OR)	95% confidence interval (CI)	β	*p*
Emotional abuse	Gender	0.444	0.313–0.629	−0.812	<.001
	Age	0.997	0.988–1.006	−0.003	.547
Physical abuse	Gender	1.114	0.81–1.530	0.108	.504
	Age	1.008	0.999–1.017	0.008	.065
Sexual abuse	Gender	0.281	0.196–0.403	−1.269	<.001
	Age	1.004	0.995–1.012	0.004	.406
Emotional neglect	Gender	0.871	0.688–1.102	−0.139	.249
	Age	1.005	0.998–1.011	0.005	.148
Physical neglect	Gender	1.103	0.908–1.340	0.098	.325
	Age	1.027	1.021–1.032	0.026	<.001

### Age differences concerning the prevalence of child maltreatment

The experience of at least one type of child maltreatment was reported most frequently in the oldest age group of 70+ (50.4%) and least often in the youngest age group of 14–19 years olds (13.4%). Participants aged between 20 and 69 years reported rather consistent rates of 24.3–33.8% (for details see Fig. 1). The largest difference between age groups was reported for physical neglect, with participants aged over 70 years reported much higher rates (46%) than participants of other age groups (for details see Fig. 1).

**Fig. 2:**
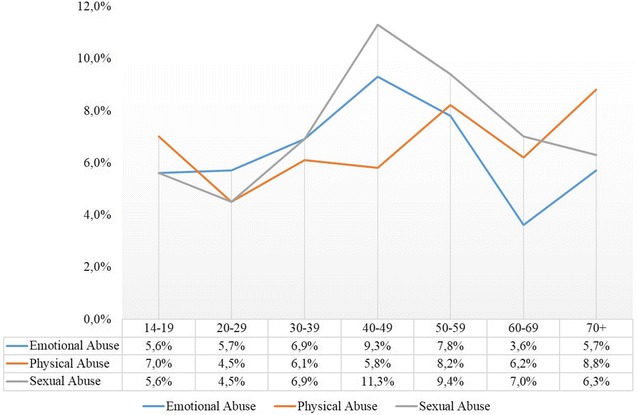
Prevalence of child maltreatment divided by age groups

Regarding child abuse, highest rates of emotional and sexual abuse were reported in the age group of 40–49 year olds. Rates of childhood physical abuse were higher in older age groups than in younger participants (see Fig. 2).

### Socio-demographic variables and childhood maltreatment

All five types of child maltreatment were analyzed separately regarding different socio-demographic variables (being employed or unemployed, level of education and equivalence income). Participants who were unemployed, had a lower level of education and a lower equivalence income reported highest rates of emotional abuse, physical abuse, and physical and emotional neglect. Sexual abuse was reported more often by unemployed participants (for details see Table 5).

**Table 5:** Prevalence of child maltreatment by socio-demographic variables

	N total	Emotional abuse N (%)	Physical abuse N (%)	Sexual abuse N (%)	Emotional neglect N (%)	Physical neglect N (%)
Employed/unemployed						
Employed	2332	144 (6.1)	150 (6.4)	172 (7.3)	304 (13.0)	520 (22.1)
Unemployed	127	19 (14.6)	17 (13.2)	17 (13.1)	26 (20.0)	38 (29.7)
Chi^2^		14.35**	8.98*	5.78*	5.30*	3.97*
Education						
Did not graduate school	55	10 (18.2)	11 (20.0)	8 (14.5)	14 (25.5)	17 (30.9)
Graduated school	2103	136 (6.5)	148 (7.0)	164 (7.8)	299 (14.2)	507 (24.1)
University degree	247	15 (6.1)	4 (1.6)	15 (6.0)	16 (6.5)	34 (13.8)
Chi^2^		11.95*	25.87**	4.55	17.80**	14.85*
Equivalence income						
<1250€/month	544	47 (8.6)	43 (7.9)	46 (8.5)	91 (16.7)	152 (27.9)
1250–2500€/month	1469	75 (5.1)	80 (5.4)	94 (6.4)	168 (11.4)	311 (21.2)
>2500€/month	392	17 (4.3)	16 (4.1)	26 (6.6)	47 (12)	62 (15.8)
Chi^2^		10.94**	6.94*	2.7	10.2**	20.7***

* p<.05, ** p<.001

## Discussion

### Prevalence rates

The aim of the present study was to provide recent prevalence data on five types of child maltreatment, assessed with the CTQ in the general population in Germany. Detailed prevalence rates are presented separately for 10 age cohorts, gender, different demographic variables and severity. Additionally, co-occurrences of and predictors for different types of maltreatment analysed. The methodology of the present study was identical to the study of Häuser and colleagues [22], who assessed child maltreatment using the CTQ in the general population of Germany. In general, prevalence rates found in the present study again underline that child maltreatment, especially physical neglect, is rather common in the general population of Germany. Overall, rates found by Häuser and colleagues, were replicated [22]. In the current sample 2.6% reported severe emotional, 3.3% severe physical and 2.3% severe sexual abuse. Additionally 7.1% reported severe emotional and 9% severe physical neglect. Compared to Häuser and colleagues [22], the rates for all types of maltreatment (except physical neglect, with 10.7), were higher: For severe emotional abuse, they reported a rate of 1.6, 2.8% for physical abuse, 1.9% for sexual abuse and 6.6% for emotional neglect. However, those higher rates might represent statistical variation and have to be examined for statistical significance. An explanation for the rise in prevalence rates may be due to an increased awareness in the general population. In 2010, right at the time, when the data collection of the study of Häuser and colleagues [22] took place, the so called abuse scandals in the Roman Catholic Church and in educational institutions with high reputations [30] came aware to the public. In its aftermath a public and political discussion about sexual abuse, but also other types of maltreatment started and a range of measures for intervention and better prevention were taken. This broad discussion might have led to a higher awareness about sexual abuse and child maltreatment in general and might have given rise to reported rates of childhood maltreatment in our study. In contrast the prevalence for physical neglect is smaller in the present study compared to Häuser and colleagues [22].

In comparison to prevalence rates for sexual abuse reported in meta-analyses, the rate of the present study for at least having experienced moderate sexual abuse is rather low [10–12]. The prevalence rate for physical neglect was higher compared to those found by Stoltenborgh and colleagues [15]. Emotional and physical neglect also have been found to be most common type of maltreatment in the US [31].

### Comparison of age cohorts

Older age was a significant predictor of physical neglect. The rates for physical neglect were the highest among those of 70 years and older with about 46%. This generation was born in, or before 1946 and therefore survived World War II and the period after the war. This period was marked by hardship for the population, thus making high rates of physical neglect not astonishing among this particular group. This age group is no longer as strongly represented in the present study as compared to Häuser and colleagues [22] due to demographic changes in the population therefore explaining lower rates of physical neglect in our sample in comparison to this former study.

Although not significantly, rates for physical abuse seemed to be declining from the oldest age cohorts towards the youngest, albeit an increase among the youngest age cohort. This general decrease from the oldest age cohorts towards those between 20 and 29 years might be due to change in norms in the society and the legal ban on physical punishment in Germany in the year 2000 [32, 33], with the higher rates in the youngest age cohort maybe explained by a clearer recall of physical abuse. For emotional and sexual abuse the pattern was quite different. For both types of maltreatment, highest prevalence rates were observed in the age group of 40–49 year olds. Quite interestingly, the present study found a steep increase in prevalence rates for sexual abuse among the age cohorts of 40–49 year olds and 50–59 year olds. However, it remains unclear whether this result represents an actual increase in rates or represents an increase in reporting due to an increased perception of the problem because of changes in social norms. The rates for emotional neglect seemed to be relatively stable across age groups. In general repeated surveys in student populations would be necessary to identify changes over time.

### Maltreatment and sociodemographic variables

Concerning other socio-demographic variables, the results generally show a higher prevalence of different types of maltreatment among those with a lower sociodemographic status. This becomes apparent in education and employment status and consequently in the monthly equivalence income, thus pointing towards the lifelong consequences of maltreatment also on the societal level that have been described in the literature [1, 5, 7, 8].

Female gender was found to be a significant predictor for sexual and emotional abuse. Especially the finding on sexual abuse is in line with findings from meta-analyses that report a preponderance for female gender [3, 10–12], and do not report a skewed distribution for the other types of maltreatment [15–17].

### Co-occurrence of different types of maltreatment

As literature demonstrates, different types of child maltreatment are interrelated and the co-occurrence of different types of maltreatment is rather the rule than the exception [34]. Due to observing a population based sample, the rates of co-occurrences of different types of maltreatment were lower than reported from clinical samples [35]. Additionally, the present study included a high number of participants reporting physical neglect without having experienced any other type of maltreatment. However, results show that both types of neglect (physical and emotional) often co-occur, as well as combinations with other types of abuse, such as emotional and physical abuse.

## Limitations

The retrospective assessment of child maltreatment may always be affected by different biases e.g. recollection biases. The random rout approach systematically excludes people that are currently residing in institutions. Therefore certain high risk-samples such as residents of child welfare institutions with a high prevalence of sexual abuse [36] may have been underrepresented in the current sample.

## Conclusions

Child maltreatment, especially physical neglect, is common among the general population of Germany. Physical neglect is highly prevalent in the (post) World War II generation and steadily declines towards the youngest age group. In general, experiences of child maltreatment are associated with a lower sociodemographic status. Women are more likely to report at least moderate levels of emotional and sexual abuse than men. Different types of maltreatment, especially physical and emotional neglect, seem to co-occur frequently.
